# Hypoxia-Inducible Factor 1-Alpha (HIF-1α): An Essential Regulator in Cellular Metabolic Control

**DOI:** 10.7759/cureus.63852

**Published:** 2024-07-04

**Authors:** Mohd Basheeruddin, Sana Qausain

**Affiliations:** 1 Biochemistry, Jawaharlal Nehru Medical College, Datta Meghe Institute of Higher Education and Research, Wardha, IND; 2 Biomedical Sciences, Allied Health Sciences, Jawaharlal Nehru Medical College, Datta Meghe Institute of Higher Education and Research, Wardha, IND

**Keywords:** targets for therapy, ldha, glut1, ros, function of mitochondria, hif-1α

## Abstract

The element that causes hypoxia when the von Hippel-Lindau (VHL) protein is not functioning is hypoxia-inducible factor 1-alpha (HIF-1α), which is the essential protein linked to cell control under hypoxia. Consequently, in situations where cells are oxygen-deficient, HIF-1α carries out a variety of essential functions. Citations to relevant literature support the notion that HIF-1α regulates the mitochondrial and glycolytic pathways, as well as the transition from the former to the latter. Cells with limited oxygen supply benefit from this change, which is especially beneficial for the inhibition of the mitochondrial electron transport chain and enhanced uptake of glucose and lactate.

During hypoxic stress, HIF-1α also controls proline and glycolytic transporters such as lactate dehydrogenase A (LDHA) and glucose transporter 1 (GLUT1). These mechanisms help the cell return to homeostasis. Therefore, through metabolic change promoting adenosine triphosphate (ATP) synthesis and reducing reactive oxygen species (ROS) creation, HIF-1α may have a role in reducing oxidative stress in cells.

This evidence, which describes the function of HIF-1α in many molecular pathways, further supports the notion that it is prognostic and that it contributes to hypoxic cell adaption. Understanding more about disorders, including inflammation, cancer, and ischemia, is possible because of HIF-1α's effect on metabolic changes. Gaining knowledge about the battle between metabolism, which is directed by HIF-1α, would help advance the research on pathophysiological situations involving dysregulated hypoxia and metabolism.

## Introduction and background

Based on this hypothesis, target genes of hypoxia-inducible factor 1-alpha (HIF-1α) were examined as the primary coordinator of cellular adaptation to the hypoxic environments and the master metabolism modulator during hypoxia. It is a component of the protean heterodimeric HIF-1, made up of HIF-1α, HIF-1β, and commercial HIF-1α. This typically happens when the body's oxygen concentration is average, and the ubiquitin-proteasome system (UPS) breaks down HIF-1α quickly [1]. On the other hand, in low oxygen environments, particularly beneath but in hypoxic conditions, after stabilizing, HIF-1α can be transported to the nucleus to dimerize with HIF-1β, in this case, to become the active transcription factor for hypoxia-responsive elements of hypoxia response elements (HREs) of genes of interest [2].

HIF-1α can function as a multifunctional supplement for hypoxia since it regulates hypoxic genes and the environment, including angiogenesis, erythropoiesis, and glycolysis [3]. This regulatory function is critical in, and not limited to, physiological conditions involving physical activities, such as exercise and acclimatization, as well as in elevated grounds or regions with high altitude, elevated terrain, or with diseases such as carcinoma, sicknesses that have inadequate blood supply, and inflammation, among others.

Among them, HIF-1α has significant implications in the case of cancer, especially in hypoxic areas within tumors, which aid in the reorganization of the metabolism in cancer cells. This reprogramming often leads to glycolysis employing a switch from oxidative phosphorylation regardless of oxygen presence, referred to as the Warburg effect [4]. This shift fosters fast proliferation and survival of the cancer cells when put in a harsh surrounding, hence promoting cancer advancement and spreading.

This review seeks to involve an extensive discussion of HIF-1α by focusing on the new research discovery that this protein serves as the critical controller of purified metabolic fractions of the cells. First, we will discuss the details of HIF-1α and how it is controlled. Second, discuss the effects of HIF-1α on any cell metabolism under different physiological and disease conditions. Third, explore the current treatment options related to HIF-1α. Thus, knowing these aspects of HIF-1α, one can glow of work done for its role in sustaining health and understanding its implication in diseases and ill health. Furthermore, it is possible to identify other research areas and clinical applications to be ventured upon.

## Review

Closely associated with the gene product is HIF-1α, which helps the hypoxic cells adapt to changed conditions during oxygen-limiting situations where cells must acquire metabolic flexibility. This one is HIF-1α, which is responsible for the transcription of several genes in a given complex by participating in various physiological and pathological processes, including angiogenesis, metabolism, and survival. To that end, the present review focuses on HIF-1α and the different metabolic shifts, emphasizing mitochondrial and glycolytic activities and their implications in multiple diseases such as inflammation, ischemia, and cancer [5].

Most physiological and various pathological processes are relatively sensitive to hypoxia, a factor that commonly influences cell condition. This adaptive mechanism involves the transcription factor known as HIF-1α, which regulates a multi-faceted response to decreased oxygen availability [6]. HIF-1α activity facilitates the maintenance of the ratio of HIF-1α to HIF-1β, which is necessary for survival and function in low-oxygen environments. HIF-1α also regulates engagement in the Warburg effect, changing the vitality and energy consumption of the cells according to the hypoxic conditions through gene regulation [7].

**Figure 1:**
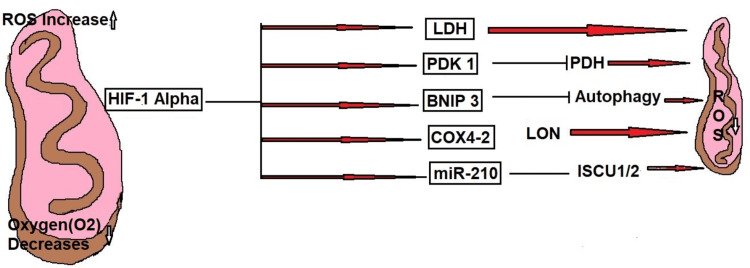
HIF-1α: suppression of the mitochondrial functions This image is the author's creation. BNIP3, Bcl-2 interacting protein 3; COX4-1, cytochrome c oxidase IV isoform 1; HIF-1α, hypoxia-inducible factor 1; ISCU1/2, iron-sulfur cluster assembly proteins; LDH, lactate dehydrogenase; miR-210, microRNA-21; PDH, pyruvate dehydrogenase; PDK1, pyruvate dehydrogenase kinase 1; ROS, reactive oxygen species

It is critical for controlling metabolism because it regulates the transition from oxygen-dependent oxidative phosphorylation to oxygen-independent glycolysis, which can be supported by the ability of HIF-1α to turn on glycolytic enzymes. This metabolic adjustment, the Warburg effect, is necessary to support adenosine triphosphate (ATP) turnover during reduced oxygen conditions [8]. Also, as indicated in Figure 1, HIF-1α suppresses mitochondrial functions, which lowers ROS production and can be a disadvantage in conditions of low oxygen availability.

There are dozens of physiological and pathological processes that are regulated by HIF-1α such as angiogenesis, cell division, survival, and metabolic flexibility. Comprehending the pro-inflammatory T-cell response and anti-tumor immunity, along with the role of metabolic regulation through HIF-1α in chronic inflammation, cancer, and ischemic diseases, is crucial for developing therapeutic approaches [9]. HIF-1α acts on metabolic pathways, and their roles in medical treatment may be established when understanding the mechanism of HIF-1α and its activities [10].

**Figure 2:**
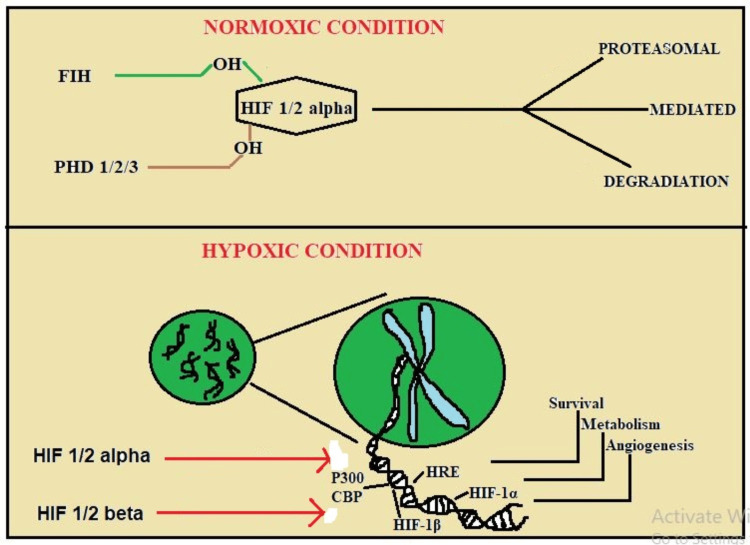
HIF-1 complex binds to normoxia and hypoxia This image is the author's creation. CBP, CREB binding protein; FIH, factor inhibiting HIF-1α; HRE, hypoxia response element; p300, cognitive potential; PHD, prolyl hydroxylase domain

The functions and regulation of HIF-1α, as well as the effects of this critical cellular player on mitochondria and glycolysis, are described with particular attention to pathophysiologic processes in diseases [3]. Understanding the exact specificities of the signaling network that is regulated by HIF-1α is our goal to come closer to understanding the roles and potential uses of this protein in the treatment. Figure 2 shows the normal state of HIF-1α, including its structure and function in the normoxia state.

HIF-1 as a transcription factor consisting of two subunits

The oxygen-stabilized form of HIF-1α is hypoxia-impaired full-length HIF-1α that is UPS and HIF-1β subunit rapidly degraded. It is unlike the situation where HIF-1α is degraded and unable to translocate into the nucleus under hypoxic conditions through which its dimerization with HIF-1β is facilitated [11]. Following up on the processes depicted in Figure 2, the transcription process is initiated once the HIF-1 complex binds to HREs provided in promoter regions of the target gene.

Structure of HIF-1α

HIF-1α and HIF-1β form an active transcriptional complex that requires dimerization (combining two similar molecules into a dimer), with the HIF-1β subunit primarily responsible for the complex's stability and functionality.

Domains Crucial for Its Regulation and Function

The least divergent protein region between plant homeodomain and basic Helix-Loop-Helix (bHLH) is the bHLH domain, which is crucial for HIF-1β interaction and DNA binding [1]. Since the bHLH domain is present, it can assist the HIF-1 complex again to select for binding to hypoxia-responsive elements or HREs that exist in some target gene promoter regions.

Per-ARNT-Sim (PAS) Domains

There are two PAS domains, PAS-A and PAS-B, which are involved in protein interactions and are described in the below sections with particular reference to HIF 1α protein. Preservation of the relationship between HIF-1α and HIF-1β would, in turn, be maintained, given that all these domains focus on the same end. The identicality has been discerned concerning interactions with other coactivators and regulatory proteins that are stable in these regions. The domain currently referred to as oxygen-dependent degradation (ODD) is responsible for regulating HIF-1α stability. When oxygen concentrations are low, the PHD enzymes add an oxygen atom, specifically hydroxyl, to specific proline residues in the ODD domain [12].

Domains at the C-terminus in Charge of Transactivation (TADs)

On HIF-1α, two TADs (TAD-N and TAD-C) are involved in HIF-1 target gene transcriptional activation, a process that occurs in these domains. Coactivators like p300/CREB binding protein (CBP) interact with TAD-C and are necessary for HIF-1α transcriptional activity [1].

Function of HIF-1α

Cell hypoxia response is governed primarily by the HIF-1α protein. This organizes the general expression of genes associated with numerous adaption processes, which is critical for cells to endure and function correctly in low-oxygen environments [4]. HIF-1α performs the following functions.

Regulation of Metabolic Pathways

Glycolysis promotion: Important glycolytic enzymes and glucose transporters have their expression upregulated when HIF-1α is present, such as LDHA, hexokinase 2 (HK2), phosphofructokinase-1 (PFK1), and GLUT1 [13]. The conversion of oxidative phosphorylation to glycolysis, or the Warburg effect, ensures that ATP is synthesized in hypoxic conditions.

Suppression of mitochondrial respiration: HIF-1α reduces mitochondrial oxidative phosphorylation to prevent the production of ROS. Pyruvate dehydrogenase kinase 1 (PDK1) is increased to achieve this. As a result, the pyruvate dehydrogenase complex is suppressed, and less pyruvate enters the tricarboxylic acid (TCA) cycle 9 [14].

Metabolic reprogramming under hypoxia

Glycolysis Enhancement

**Figure 3:**
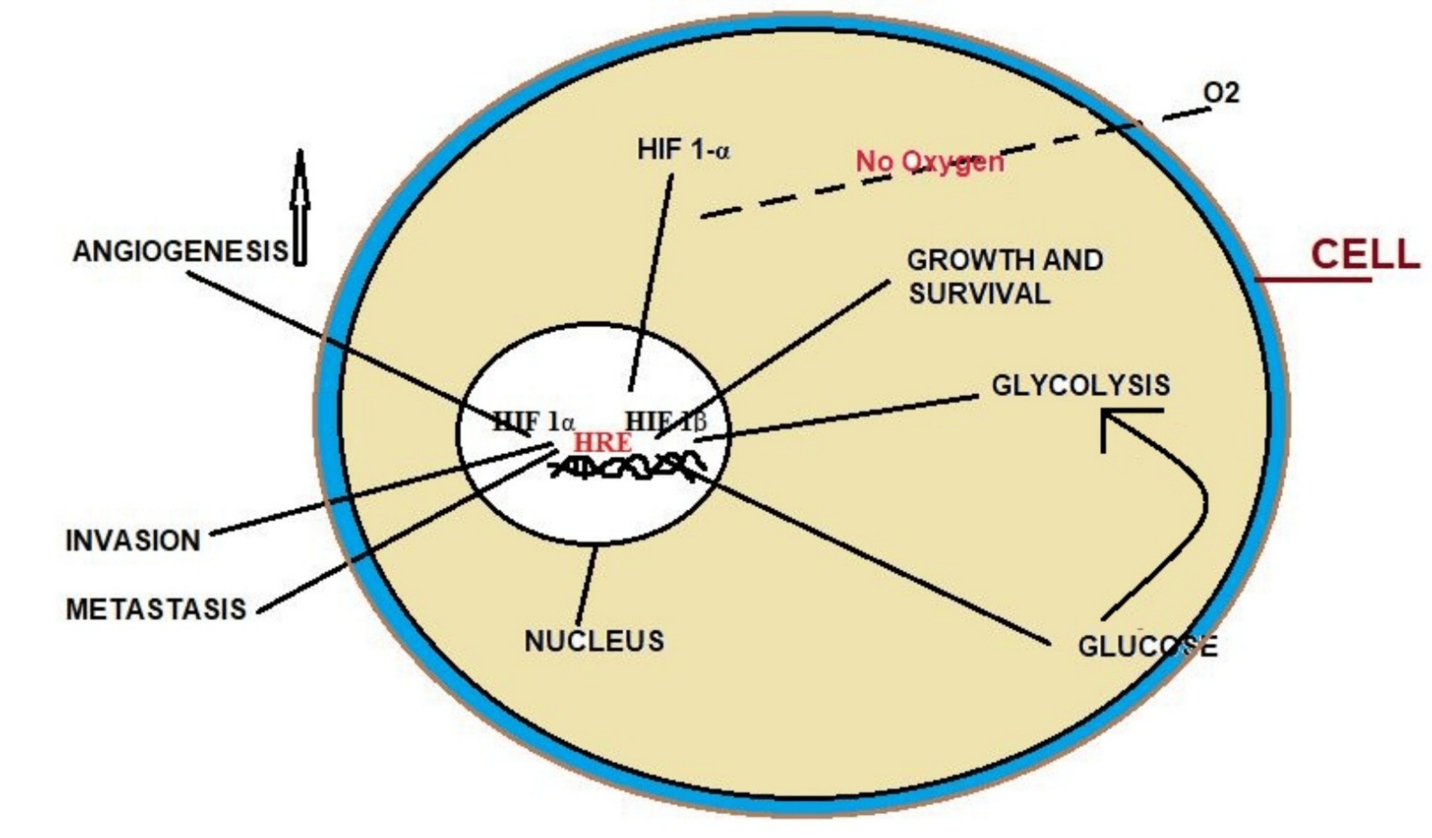
Glycolysis: HIF-1α up-regulate the expression of glucose This image is the author's creation. HRE, hypoxia response elements; HIF-1α, hypoxia-inducible factor 1-alpha

Metabolic reprogramming is a crucial adaptive mechanism cells undergo to survive and function in hypoxic settings. HIF-1α is essential for regulating the metabolic shift because it allows cells to continue producing energy and lessen oxidative damage. This study explores how HIF-1α affects mitochondrial and glycolytic pathways, promoting cellular adaption to low oxygen levels [7]. HIF-1α is involved in the translocation of the cells back to glycolysis upon hypoxia from oxidative phosphorylation [8]. During conditions of low oxygen availability, there is something known as the Warburg effect, which is a fundamental change in how ATP is produced. The HIF-1α up-regulates the expression of glucose transporter 1, allowing more glucose quantum to be transported into the cell [15]. This helps to ensure that glycolysis will have enough glucose to work with, as shown in Figure 3. Further, HIF-1α up-regulates the expression of several crucial enzymes involved in glycogen content and the glycolytic enzyme complex. One such enzyme is HK2, which is insignificantly implicated in converting glucose to glucose-6-phosphate and opens the pathway to glycolysis [16].

PFK1: The control of glycolysis, therefore, relates to regulating the constriction that transforms fructose-6-phosphate into fructose-1,6-bis phosphate, which is a rate-controlling step.

LDHA: Generates lactate from pyruvate, resupplying NAD+ required for ongoing glycolytic activity.

Increased generation of lactate: HIF-1α promotes the expression of LDHA, which prevents pyruvate from building up and makes it easier for pyruvate to be converted to lactate, ensuring the continuation of glycolysis. Lactate, produced in cells, is pumped out through monocarboxylate transporters (MCTs), which are also regulated by HIF-1α [17].

Mitochondrial Activity Suppression

HIF-1α concurrently inhibits mitochondrial oxidative phosphorylation to counterbalance the rise in glycolysis. To lessen the generation of ROS, which can be harmful in hypoxic environments, essential strategies include the following.

Pyruvate dehydrogenase (PDH) inhibition: HIF-1α induces expression of PDK1 phosphorylated PDH and inhibits its activity. It regulates pyruvate’s entry into the mitochondria and its conversion to acetyl-CoA, which reduces its ability to be processed by the TCA cycle [14].

Promotion of mitophagy: The levels of the latter are increased, and regeneration of PDK1 upon HIF-1α is induced. PDH phosphorylation is also inhibited. Today, it helps to avoid the TCA cycle by not converting pyruvate to acetyl-CoA too often.

Suppression of mitochondrial biogenesis: HIF-1α represses the expression of essential regulators of mitochondrial biogenesis, such as PGC-1α (peroxisome proliferator-activated receptor gamma coactivator 1-alpha), which is an example of inhibiting choroid metabolic syndrome (CMS) from forming new structures [18].

Integration of Metabolic Pathways

The pentose phosphate pathway (PPP): HIF-1α also increases the expression of glucose-6-phosphate dehydrogenase (G53PD), which pushes the PPP to its limit [19]. Here, in this process, the required compound called ribose-5-phosphate for nucleotide synthesis and another important compound called NADPH to avoid oxidative stress are synthesized. HIF-1α also increases the expression of glucose-6-phosphate dehydrogenase (G53PD), pushing the PPP to its limit [19]. Here, in this process, the required compound called ribose-5-phosphate for nucleotide synthesis and another important compound called NADPH to avoid oxidative stress are synthesized.

Lipid metabolism: By controlling the genes involved in producing and oxidizing fatty acids, HIF-1α influences how fat is metabolized. When oxygen levels are low, the modification is essential for maintaining a cell's energy reserves and creating cell membranes. HIF-1α controls how glutamine is used and other elements of amino acid metabolism. Within the TCA cycle, glutamine can be converted into α-ketoglutarate, which enhances metabolic processes and speeds up energy creation [20].

Mechanisms at the molecular level and networks of regulation

The transcription factor 1-alpha (HIF-1α) is an essential element that triggers hypoxia by controlling how cells react to reduced oxygen levels. Precise mechanisms control complex molecular networks' activity, location, and stability. Understanding these regulatory networks is critical to understanding how HIF-1α regulates the cellular response to hypoxia and to identify possible targets for treatment in hypoxic diseases.

Oxygen-Dependent Regulation

**Figure 4:**
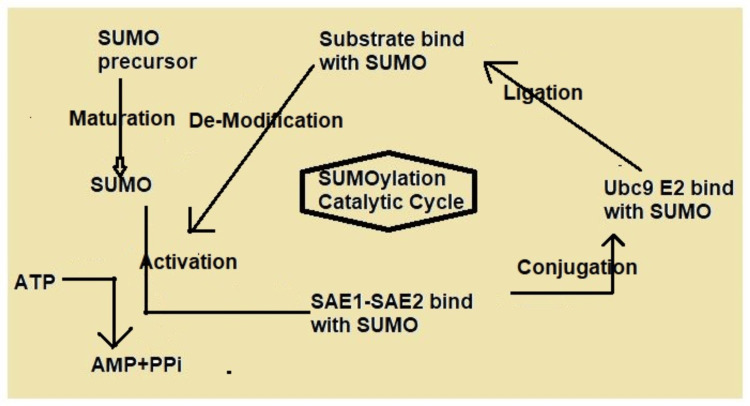
SUMOylation cycle This image is the author's creation. AMP, adenosine monophosphate; ATP, adenosine triphosphate; PPi, proton pump inhibitor; SAE1, anti-small ubiquitin-like modifier 1-activating enzyme; SAE2, anti-small ubiquitin-like modifier 2-activating enzyme; SUMO, small ubiquitin-like modifier; Ubc9 E2, ubiquitin-conjugating enzyme E2

Oxygen levels primarily control HIF-1α stability through the actions of prolyl hydroxylases (PHDs) and the von Hippel-Lindau (VHL) protein. In normoxic conditions, PHD can modify HIF-1α through regulation because of proline residues at positions 402 and 564 located in a region called the ODD domain [12]. Following the identification of hydroxylated HIF-1α, the target VHL protein initiates the ubiquitination and proteasomal degradation of the HIF molecule, as illustrated in Figure 4. Factor-inhibiting HIF (FIH) also oxidizes at an asparagine residue in the C-terminal trans-activation domain (TAD-C) of HIF-1α. HIF-1α cannot activate under normoxic settings because specific inhibitors prevent it from attaching to p300/CBP coactivators.

Post-Translational Alterations

Several post-translational modifications (PTMs) further control HIF-1α (phosphorylation). The stability and function of HIF-1α are impacted when kinases in pathways, including PI3K/AKT/mTOR and MAPK, phosphorylate the protein [21]. For example, mTOR signaling upregulates HIF-1α expression and stabilizes its distribution under hypoxic conditions. The ability of HIF-1α to be stable and to transcribe involves the processes of acetylation and deacetylation with acetyltransferases like p300/CBP and deacetylases like SIRT1 transforming growth factor-beta is a potent angiogenesis inhibitor; it requires oxygen to be stable and to function [22].

SUMOylation: As demonstrated in Figure 4, SUMOylation may affect the stability and function of HIF-1α, with different consequences based on the cellular environment.

Co-repressors and co-activators

This can, however, be attributed to the fact that the interaction of HIF-1α with DNA is associated with its ability to recruit corepressors as well as coactivators.

p300/CBP

Co-activators are necessary for excluding and including the transcriptional components at the target promoter of the genes and for enhancing the HIF-1α’s transactivation [23]. HIF-1α’s stability and transactivation ability are modulated by binding to scleroderma renal crisis (SRC-1) or tafazzin (TAZ) proteins.

Corepressors

FIH acts as a VHL-homologous protein that occupies ubiquitin-cyclin-dependent kinase, hence inhibiting the binding of HIF-1α to its coactivators in normoxic conditions [24].

Signaling Pathways

HIF-1α activity is controlled by several intracellular signaling pathways, including phosphosphoinositide 3 kinase (PI3K)/Akt/mammalian (or mechanistic) target of rapamycin (mTOR) AKT/mTOR/PI3K pathway. This route facilitates translation and prevents degradation, hence improving HIF-1α stability and efficacy. Creating the HIF-1α protein depends on the healthy operation of mTOR signaling [25].

Mitogen-activated protein kinase/extracellular signal-regulated kinase (MAPK/ERK) pathway. HIF-1α's transcriptional activity is increased when ERK phosphorylates it under hypoxic conditions [26].

AMP-activated protein kinase (AMPK) pathway: AMPK typically reduces mTOR signaling in low-energy conditions, which affects HIF-1α.

Crosstalk with other hypoxia-inducible factors

HIF-1α can modify its activity and the overall hypoxia response through interactions with other members of the HIF family, including HIF-2α and HIF-3α. These interactions might be either hostile or cooperative depending on the particular cellular environment and HIF isoforms involved [27].

MicroRNA (miRNAs) Regulation

MicroRNAs (miRNAs) can post-transcriptionally regulate HIF-1α [28].

miR-155: Increases the activity of HIF-1α by down-regulating the elements that encourage its breakdown.

**Figure 5:**
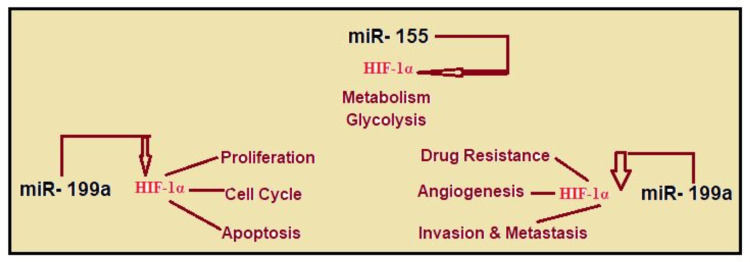
MicroRNA regulation This image is the author's creation. HIF-1α, hypoxia-inducible factor 1-alpha; miR, microRNA

miR-199a: Reduce HIF-1α mRNA expression and activity by directly targeting it (Figure 5).

Implications for disease

HIF-1α-driven metabolic reprogramming has significant consequences for several illnesses.

Cancer

Hypoxic microenvironments in tumors frequently result in HIF-1α activation. Rapid cell growth and survival are supported by the switch to glycolysis and the reduction of mitochondrial activity. Cancer therapy may involve targeting HIF-1α or its metabolic pathways [29].

Ischemic Diseases

HIF-1α-mediated metabolic adaptation enables cells to withstand low oxygen levels, such as myocardial infarction and stroke. In these circumstances, results could be improved by increasing HIF-1α activity or imitating its effects [30].

Chronic Inflammation

The metabolic adaptability of immune cells is influenced by HIF-1α, which affects the cells' activity and inflammatory responses. Our expanding knowledge of the function of inflammatory illnesses could pave the way for novel therapeutic options [8].

Therapeutic potential

Cancer Therapy

**Figure 6:**
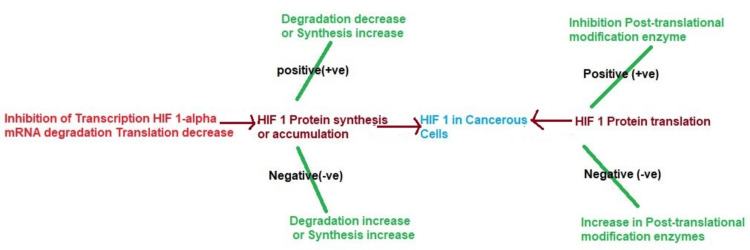
HIF-1α in cancerous cells This image is the author's creation. HIF 1, hypoxia-inducible factor 1

HIF-1α is mainly overexpressed in solid tumor types, where it, therefore, has a dual role in promoting angiogenesis, metastasis, and resistance to treatment, which are all critical in tumor progression. Cancer treatment is depicted in Figure 6, and strategies aim to target HIF-1α to address these factors specifically.

Repression of HIF-1α expression: Small interfering RNAs (siRNAs) or antisense oligonucleotides can be utilized to specifically block HIF-1α expression, leading to reduced levels and impeded tumor growth [31].

Inhibitors of HIF-1α: siRNAs or antisense oligonucleotides can be utilized to specifically block HIF-1α expression seen in Figure 6, leading to reduced levels and impeded tumor growth.

Targeting HIF-1α downstream pathways: The suppression of tumor growth and improvement in treatment outcomes can be achieved by inhibiting HIF-1α-regulated genes involved in angiogenesis (e.g., vascular endothelial growth factor (VEGF) inhibitors) or glycolysis (e.g., glycolytic enzyme inhibitors) [32].

Ischemic Diseases

Tissue damage and dysfunction can occur as a result of ischemia-reperfusion injury in diseases like myocardial infarction and stroke [33]. Treatment strategies for ischemic conditions frequently focus on HIF-1α as a critical target, and treatment strategies for ischemic conditions frequently focus on HIF-1α as a crucial target:

HIF-1α stabilizers: Stabilization of HIF-1α can be done with the help of PHD enzyme inhibitors or small molecules that replicate hypoxia. Through this technique, cells repair tissues, while HIF-1α is promoted to defend against ischemia damage [34].

Gene therapy: HIF-1α or any of its downstream target genes may be delivered using viral vectors or other forms of gene delivery, using tissue-specific promoters to promote expression of the transduced genes in ischemic tissues with the overall goal of promoting angiogenesis and cell survival.

Metabolic regulators: HIF-1α regulated metabolic pathways, such as glucose utilization (glycolysis) and mitochondrial energy generation, might benefit from drugs that ease tissue repair and protect cells from ischemia [35].

Inflammatory Diseases

Therapies aimed at HIF-1α-regulated metabolic pathways, including glycolysis and mitochondrial activity, may shield cells from ischemia injury and encourage tissue regeneration. Therapies aimed at HIF-1α-regulated metabolic pathways, including glycolysis and mitochondrial activity, may shield cells from ischemia injury and promote tissue regeneration.

HIF-1α activators include respective chemicals that activate or maintain HIF-1α and can lessen inflammatory processes [36] since they suppress cytokines production that promotes inflammation and promote cytokines production that resolves inflammation

Anti-inflammatory drugs: By stabilizing or activating HIF-1α, the healing of inflammation and suppressing the liberation of pro-inflammatory cytokines may reduce inflammatory reactions [37].

Immune system modification: Altering the level of HIF-1α expression or activity could also impact the character and function of immune cells - something that could need to be done to treat some autoimmune diseases or some conditions with incorrect immunological activity [38].

Challenges and future directions

Targeting HIF-1α has the potential to treat a variety of illnesses. However, there are still a few obstacles that need to be cleared. This made focusing on HIF-1α have the potential for the treatment of numerous diseases.

Particularity and Off-Target Impacts

To cut on toxicity and collateral effects and also to affect other HIF isoforms or other physiological processes, this HIF-1α should be targeted selectively.

Medication Administration

Using small molecules as HIF-1α-targeting drugs requires selective delivery of these drugs to the target tissues or cells, which becomes a challenge if it is systemic administration for diseases such as cancer or ischemic diseases [5].

Resistance Mechanisms

It, therefore, implies that through the use of one path or the other, various routes or compensatory mechanisms may make cancer cells and other sick tissues insulated from HIF-1α-targeting therapy. Thus, by escalating knowledge on HIF-1α control, increased literature about HIF-1α control and influence can lead to the development of improved treatment for ischemic diseases, inflammatory diseases, and cancer. While there are currently many barriers, it has been demonstrated that examining the possibility of focusing on HIF-1α and its therapeutic potential reveals the prospect of building more effective and individualized treatment protocols [39].

## Conclusions

In summary, HIF-1α plays a crucial role in regulating various cellular functions and facilitating the body's ability to adjust to low oxygen levels. It is triggered in low oxygen tension environments and results in the production of several genes that guarantee cell survival in hypoxic stress situations. They are also connected to glucose uptake, blood vessel development, and cell survival. This is only one aspect of HIF-1α activity; it also summarizes data from other metabolic pathways to ensure proper, balanced operation of cells involved in energy production and, consequently, energy expenditure. Because of its significant metabolism-regulating abilities, HIF-1α has the potential to be a therapeutic target for a number of illnesses and ailments, such as cancer, ischemia, chronic inflammation, and any other pathologies caused by metabolic disturbances. Analyzing HIF-1α in its dual functions of defining degradation and pro-survival offers valuable insights into the general ideas of cellular adaptability and opens up new avenues for therapeutic intervention.
